# Quantum Dots: An Emerging Tool for Point-of-Care Testing

**DOI:** 10.3390/mi11121058

**Published:** 2020-11-29

**Authors:** Suchita Singh, Aksha Dhawan, Sonali Karhana, Madhusudan Bhat, Amit Kumar Dinda

**Affiliations:** Department of Pathology, All India Institute of Medical Sciences, New Delhi 110029, India; suchitaasingh@gmail.com (S.S.); akshadhawan@gmail.com (A.D.); karhanasonali@gmail.com (S.K.); bhat.madhusudan86@gmail.com (M.B.)

**Keywords:** microsystem, quantum dots, point-of-care testing

## Abstract

Quantum dots (QDs) are semiconductor crystals in the nanodimension having unique optical and electronic properties that differ from bulk material due to quantum mechanics. The QDs have a narrow emission peak, size-dependent emission wavelength, and broad excitation range which can be utilized for diverse biomedical applications such as molecular imaging, biosensing, and diagnostic systems. This article reviews the current developments of biomedical applications of QDs with special reference to point-of-care testing.

## 1. Introduction

Microsystems (MS), an emerging discipline, initially known as micro-electro-mechanical systems, are also denoted as micromachines, microdynamics, microrobots, etc. [1]. In the last 20 years or so, this field has hugely expanded into a big, high-tech, international domain whose importance and potential for the present and future are undisputed. The main characteristic of microsystems is their capability to make a single system miniature particles or devices of characteristic sizes ranging from few nanometers to millimeters. They have made huge clinical impact in the field of therapeutics and clinical diagnostics, especially in the area of point-of-care testing (POCT) [2]. They have the potential to acquire data from their environment by making standalone decisions while being able to influence their environment. In addition, they allow achieving many diagnostic procedures, interventional procedures, and sophisticated tests in low-cost settings. Due to these features, they have huge potential to have a significant impact on healthcare, and they are suited for use in various biomedical applications [3]. Microsystem-enabled healthcare systems can not only benefit developed nations by providing innovation in healthcare, but also allow expansion of advanced healthcare concepts to developing nations. Thus, the concept of microsystems is likely to create new generations of POCT helping early diagnosis, allowing determination of prognosis, reducing morbidity, and improving therapeutic outcomes; alternatively, they can be applied for entirely new indications [4].

Considering the high importance of microsystems in point-of-care testing, in this Special Issue, we review the emerging field of quantum dots (QDs), and we provide an overview of their significant advancement and application as microsystems. Here, it is important to emphasize that there is a significant knowledge gap regarding QDs and their effects within the field of point-of-care testing. We, therefore, draw upon what is known about various types of QDs and their clinical utilities to generate hypotheses that could drive future research and benefit technologically applied fields. Certainly, our focus on QDs does not rule out the use of several other potential microsystems for POCT. These important topics are reviewed in detail elsewhere in this Special Issue.

### 1.1. Quantum Dots

Quantum dots are solid semiconductor nanostructures, having a particle size of less than 100 nm; they are currently of great interest. They have the potential to extend across volume and molecular levels, thereby opening new approaches, especially in biological research, the biomedical and pharmaceutical industries, and healthcare [5,6,7]. QDs were first synthesized by Louis E. Brus in the 1980s and attracted interest from various fields [8]. These particles exhibit unique optical and electronic properties as they have a small size along with a high surface area, and they show interesting phenomena such as a narrow emission peak, size-dependent emission wavelength, and broad excitation range [9,10,11]. Nanostructures can be of different dimensions, such as quantum wells with two dimensions, quantum wires with one dimension, or quantum dots with zero dimensions [12]. Semiconductor quantum dots are crystalline molecules made up of hundreds to millions of atoms with only a small number of free electrons. Colloidal semiconductor QDs are visible luminescent nanomaterials synthesized mainly with atoms of groups II–VI, III–V, or IV–VI, among which group II–VI compounds such as CdSe, CdS, and CdTe have gained importance, especially in medical applications, as they are the most widely developed and utilized [13]. As the sensitivity of QD detection is higher than that in other organic dyes, they have become attractive fluorophores, thus facilitating cellular imaging and quantitative cellular analysis with the help of fluorescent probes. In addition, their surface modification is easily achievable with antibodies or DNA [14]. Furthermore, QDs can also be prepared using the same material while producing different emission colors by just changing their size [15]. Overall, the volume of research related to QDs has increased significantly with their incorporation in various innovations leading to commercialization efforts. In the present review, we succinctly discuss the properties, synthesis, fabrication, and application of QDs in point-of-care testing.

### 1.2. Properties of Quantum Dots 

As quantum dots are essentially semiconductors, they are also known as semiconductor nanocrystals. Since they are significantly smaller in size than bulk semiconductor materials and slightly bigger than individual atoms, QDs exhibit properties that seem to be somewhere between bulk materials and single atoms. Due to their unique size, quantum effects govern the properties of quantum dots. 

Electrons exist in discrete energy levels in a bulk semiconductor. The lower energy level is termed as the valence band, whereas the higher energy level is known as the conduction band. The energy difference between both bands is termed the band gap. An electron can jump from the valence band to the conduction band by absorbing energy, e.g., light or heat. As the electron leaves the valence band, it leaves a hole behind. The hole and electron together constitute an exciton. The average distance between an electron and a hole is called the exciton Bohr radius. When the size of a semiconductor falls short of the Bohr radius, it becomes a quantum dot and experiences quantum confinement [16]. Confinement here refers to the fact that the random movement of electrons is restricted or confined in QDs. This results in the formation of more discrete energy levels. As we decrease the size of particles, the energy levels become more discrete and, in turn, increase the band gap. This increase in band gap also results in enhanced gap energy. Therefore, the size of a crystal can determine the band-gap energy, which in turn determines the energy of the emitted photon [17,18]. QDs have a large absorption spectrum [19], i.e., they can be excited by a large range of wavelengths; furthermore, since their size controls the energy of the released photon, the emission spectrum can be restricted to be as narrow as possible by the user. Small-sized QDs exhibiting blue color have a larger band gap, while red QDs have a smaller band gap.

### 1.3. Synthesis and Fabrication of Quantum Dots 

Various types of QDs are synthesized like ZnS, ZnSe, CdSe, CdS, CdTe, PbSe, PbS, and out of which CdSe/ZnS core/shell QDs are often used in the application of biological importance [20]. The emission wavelength of QDs ranges from ultraviolet to the infrared. Some of the QDs are comprised of toxic ions like CD^+2^, Se^–2^, and Te^–2^; thus, coating based on oxides is required to minimize biological applications toxicity [21,22,23,24]. Quantum dots that are generally applied to biomedical applications are made such that they have a core of a specific semiconductor material such as CdSe, which is then shelled by a different semiconductor material like ZnS. This ensures QDs to manifest better optical properties [25]. The synthesis of core and subsequent shelling can be done in three ways, which forms three types of QDs, i.e., type I [26], reverse type I [27], and type II [28]. In type I, the band gap of core material is smaller than the band gap of the shell material, with both the holes and electrons confined in core itself. The shell isolates the core from the medium surrounding it, which facilitates less interference by oxygen and water molecules. This prevents any changes in optical properties of QDs induced by oxygen and water molecules. The most widely used type I core/shell QD is CdSe/ZnS [29], in which ZnS shell improves the stability against photo-degradation [30] and the optical properties of the QDs [5]. In reverse type I, the band gap of the core material is larger than the band gap of the shell material, wherein the holes and the electrons may be partially confined or completely confined in the shell. In this type, the emission wavelength is governed by the thickness of the shell. CdS/HgS [31], ZnSe/CdSe [32], and CdS/CdSe [33] QDs are the most extensively studied systems. These QDs, however, have to be saved against photo-degradation, which can be done by adding onto a second shell with a larger band gap semiconductor material. In type II, the valence band or the conduction band of the shell is placed at band gap of the core material, which results in a staggered alignment. The resulting alignment is smaller than that of the original semiconductor materials used for developing the core and the shell of the QDs. This type is basically an attempt to modulate the size and therefore the energy of the emitted photon more precisely. Type II have been synthesized for infrared applications [28] and the most widely applied are CdSe/ZnTe and CdTe/CdSe.

Generally, two types of approaches are used to fabricate the QDs. These can be classified as a top-down approach and bottom-up approach, which is briefly described below. 

#### 1.3.1. Top-Down Approach

Varying methods have been adopted for top-down processing that include ion implantation, e-beam lithography, molecular beam epitaxy (MBE), and X-ray lithography in which QDs are formed by thinning of bulk semiconductors. In these methods, the bulk semiconductor is thinned which results into the formation of QDs. Electron beam lithography, focused ion, and wet chemical techniques are used to form the QDs of diameter ~30 nm from the bulk semiconductor materials. To achieve the desired packing geometries with the controlled shapes and sizes, there is a requirement of systematic experiments on quantum confinement effect. However, there are certain limitations of using these processes like the incorporation of impurities during the synthesis process, size non-uniformity and structural imperfections by patterning [34].

Electron Beam Lithography:

Surface having the electron sensitive film called resist are used to draw the customized shapes by the scanning of the focused beam of electrons. The resist is composed of the polymeric compound and is called negative tone when it is composed of long chain polymer having a high molecular weight and positive tone when it is composed of short chain polymer. In the developing process, when the resist is immersed in the solvent, either the exposed or covered part is removed as the electron beam alters their solubility. Therefore, the small structures that are created in the resist can be transferred to the substrate by the process of etching. This method provides a high degree of flexibility in the nanostructures having sub 10 nm resolutions [35].

Focused Ion Beam Technique:

In this, a finely focused beam of ions is employed that can be used for imaging or site-specific sputtering/milling process when operated at low or high voltage respectively. Depending on the ion beam size, the size, shape, and the inter-particle distance can be controlled. Quantum dots of 8–20 nm diameter formed by this technique have been reported [36].

Etching Techniques:

Etching is also an important step for the process of nanofabrication, which consists of application of a radio frequency voltage to create etching chamber reactive gas species and plasma. It further helps in breaking down the gas molecules into more oxidizable fragments and produces volatile reaction by-product to etch different shape of the sample. However, these are time-consuming and expensive processes [37].

#### 1.3.2. Bottom-Up Approach

In this approach, variety of self-assembly techniques is used to fabricate the highly crystalline narrowly dispersed QDs. These can be categorized as wet-chemical and vapor-phase methods [35]. 

Wet Chemical Methods:

It includes sol–gel, micro-emulsion and the hot solution decomposition and follows the conventional precipitation methods including, a single or mixture of solutions with the controlled parameters. The precipitation process involves nucleation that can be homogeneous, heterogeneous or secondary nucleation and limited growth of nanoparticles [38,39,40]. In the hot-solution decomposition process, QDs are fabricated at high temperatures with the help of the organometallic precursors, growth solvents, or ligands like trioctyl phosphine or trioctylphosphine oxide that stabilizes the QD dispersion and helps in improving the passivation of the surface [41]. This synthesis process is facile and can be done in ‘one-pot’. It produces enough thermal energy that can normalize the defects and thereby helps in monodispersed fabrication, and large quantities of QDs [42] along with the alloying process [43]. CdSe/ZnS core/shell is commonly utilized in the biomedical approach in which the CdSe core is over coated with ZnS shell, which has a wider bandgap. This coating helps in passivizing the surface as well as stabilizing the photophysical properties of CdSe core QDs [34].

Vapor Phase Methods:

These include molecular beam epitaxy (MBE), physical vapor deposition (PVD), and chemical vapor deposition (CVD) [44]. In this method, with the help of atom-by-atom process, the layers of quantum dots are grown without any patterning. Interfacial energies and the lattice mismatch helps in their growth. This method results in the effective production of the quantum dot arrays in the absence of a template, however, in homogeneous optoelectronic properties have been observed due to the variations in the size of quantum dots [45,46,47,48].

#### 1.3.3. Other Synthetic Methods

Other methods for the QD synthesis include ultrasonic or microwave irradiation, hydrothermal synthesis, from microorgansim bio-template and electrochemical assembly. In ultrasonic or microwave irradiation, synthesis of QDs of diameter 1–5 nm is done by passing the ultrasonic or microwaves in the aqueous medium containing the precursors under the inert conditions [49,50]. In the hydrothermal method, the crystallization of the inorganic salts is done from aqueous solutions, which are controlled by pressure and temperature [51,52]. Bio-templates from the microbial origin are also used for the fabrication of QDs using bottom-up approach resulting in the cost-effective, quick growth and controllable size and structure of QDs [53]. For example, genetic engineered phages can be used like M13, Fd that can recognize the specific semiconductor surface by combinatorial phage display [54,55]. In the electrochemical technique, the ionic reaction at an electrolyte–metal interface can result in the self-assembly of QDs onto the metal [56,57].

#### 1.3.4. Surface Coating and Functionalization of QD

Once the QDs are synthesized, they are hydrophobic in nature. Hydrophilization of these QDs is a prerequisite for their exploitation in biological systems. Only when they are coated with polymers, that render them hydrophilic nature, will the QDs be conjugated to various types of bio-molecules like peptides, antibodies or ligands. Other than water solubility, the properties required by QDs for their biomedical applications are high stability, resistance against photo-degradation, smaller size, and presence of functionalized groups on their surface. Each of these parameters can be controlled as per the needs of the user. The strategies used to impart water solubility to QDs can be broadly divided into two approaches, the first one replaces the ligands acquired by the QDs during the time of synthesis and the second approach involves simply capping the ligands over by any amphiphilic polymers. Both the approaches have their own pros and cons, and the one that the user chooses depends on the specific application where the QDs have to be employed. When amphiphilic groups exchange the original hydrophobic ligands, the diameter of the quantum dots is not affected but it affects the quantum yield and optical properties of the QDs. The most popular strategy for ligand exchange is the introduction of thiol groups, like dithiols or thiol dendrimers. Thiolated polyethylene glycol (PEG) polymers are being used extensively as they are quite easy to handle and process [58,59,60,61]. Another technique for ligand exchange is grafting dendrimers. Dendrimers are mono-dispersed and highly branched molecules, which can be functionalized onto QDs. One such dendrimer is poly (amidoamine) (PAMAM) [60,61], which has the property of penetrating into cell walls [62]. Various work groups have also attached only amine groups onto the surface of QDs with the use of poly (ethyleneimine) [63]. Amine functionalization is also done using poly (N, N-dimethylamino-ethyl methacrylate), which shows better colloidal stability in biological systems [64]. 

The second approach involves capping the original ligands by amphiphilic polymers or encapsulation. The common property of all these polymers is that they all possess a lipophilic part, which intercalates between the original ligands, or they simply encapsulate the quantum dot leaving the ligands still in their original position. Widely used amphiphilic polymers are polyacrylic acid [65] and maleic (anhydride-alt-1-tetradecene) [66], which could be further linked with diamine to impart better stability. Quantum dots are also encapsulated within micelles because it comes with the advantage of availability of a wide variety of surfactant/lipids with various terminating groups. This method ensures that the optical properties of the encapsulated QDs are preserved and offers higher biocompatibility and low cytotoxicity. Mostly, PEG derived phospholipids are used for encapsulation as it imparts better water-solubility [67,68]. 

As for the biomedical applications, QDs are used in solution or colloidal form. They need to be water-soluble and biofunctionalized for the long-term stability in water as well as for incorporating with other functional groups for the bioconjugation. They can be made water-soluble through ligand exchange or by native surface modification. Since the monodispersed QDs are synthesized at high temperature in the organic solvent and in the presence of surfactants, the fabricated QDs are surfactant coated. The polar head group of the surfactant is attached to the inorganic surface whereas the hydrophobic chain faces towards the organic solvent. In order to be water-soluble, the surfactant layer is either replaced or coated with additional hydrophilic polymers. Another way to introduce hydrophilicity is by the exchange of the surfactant coating with the molecules, which has two functional groups, one group like thiol or carboxyl, which reacts towards the nanoparticle surface, and the other has hydrophilic moiety [69].

Once the QDs are made water soluble, then they can be conjugated to biomolecules so that they can function at specific targets in a biological system. These biomolecules generally include proteins, peptides, antibodies, or certain ligands. The amine, carboxyl, and the sulfhydryl groups of these biomolecules are targeted for their conjugation with the QDs. Glutaraldehyde conjugation is an amine to amine conjugation wherein amine group of the protein or peptide conjugates with amine group on QDs, with glutaraldehyde acting as a linker between the two. Glutaraldehyde can also be replaced by disuccinimidyl suberate (DSS) [70]. Another method that targets amine groups of biomolecules is the Diketone amine chemistry, wherein acetoacetoxy groups on QDs can directly react with amine groups on antibodies or any protein molecule [71]. A popular method that targets the carboxyl groups of proteins is the EDC-NHS chemistry [72,73]. However, the drawback of all the above-mentioned procedures is that they are all unspecific as a result of which the site of the conjugated molecules required for further application might not be ajar for use. This drawback is rectified by using strategies that target sulfhydryl groups, like the maleimide conjugation in which the crosslinker sulfo-SMCC (sulfosuccinimidyl 4-(N-maleimidomethyl)-cyclohexane-1-caroxylate) contains a maleimide group that reacts with free sulfhydryls on the protein or antibodies. By reacting the reagent first to the QDs (with its numerous amines) and then to a peptide containing a reduced terminal cysteine, all peptide molecules can be conjugated with the same predictable orientation. Similarly, PMPI (N-(p-maleimidophenyl) isocyanate) can bind to silica coated QDs which then acts as a linker between the QDs and sulfhydryl groups of proteins [74]. Devising strategies for the conjugation of biomolecules to QDs is presently a wide area of research and many groups are working on finding out the best technique for bioconjugation. Easier methodologies will pave the way for manufacturing novel bio-conjugated nano-constructs, thus leading to new and efficient point of care devices.

## 2. Advantage of QDs for Fluorometric Testing Over Fluorescent Dye Based Methods

Majority of the fluorescent or organic dyes have several limitations like they are more prone to photobleaching, shows self-quenching at high concentrations, they are pH dependent and unstable in aqueous solutions for long-term [75]. As compared to other fluorescent or organic dyes, QDs have unique properties due to which they are preferred for fluorometric testing. These include unique optical and electronic properties due to their reduced size, tunable size dependent fluorescence emission, broad excitation due to which they can be excited with multiple fluorescence colors simultaneously at a single wavelength with narrow and symmetric emission spectra. They can be synthesized to be effectively monodispersed with improved emission intensity, large absorption coefficients, and excellent resistance against photobleaching [9]. QDs are used as fluorescent probes using the process of bioconjugation which involves linking two biomolecules together through covalent linkage. They can be utilized in the process of cellular and molecular labeling, single cell surface receptor imaging, in vivo and in vitro imaging, and other biosensing applications like immunoassays, non-invasive detection of small tumors, detection of nucleic acid, proteins and sugar, clinical laboratory diagnostics, and resonance energy transfer studies [76].

## 3. Point-of-Care Testing

In a current shifting patterns POCT should be perceive as a broader spectrum of technologies (simplest to more advanced), settings (hospitals, homes, clinics, communities and peripheral laboratories), and users (highly trained to lay person). Otherwise in a standard definition, it is a clinical diagnostic laboratory testing designed to be used at the point in time, performed at or near the clinical laboratory site and could be in closed proximity where the patient is located and receiving treatment care [77,78,79]. The testing and care is typically conducted by clinical personal or self-testing by the patients itself. Point of care testing is otherwise determined as any testing performed bedside, near patient, outside of the conventional, core, or central laboratory usually performed by non-laboratory staff and the outcomes are utilized for the clinical decision making [80,81]. Their major objectives are to generate quick results so that suitable treatment can be executed, which can lead to enhancing clinical and economic outcomes [82,83]. POCT has a variety of procedures and complexity that differ from manual procedures to automated analyzers [84].

### 3.1. Point-of-Care-Testing: A Global Need

The necessity to create healthcare that is patient-centered rather than the provider is now a global trend [85]. The advancement has expanded from traditional formats which require expensive care, centralized testing, more than one visit to the healthcare for complete assessment process, to newer platform dependent on handheld devices [86,87,88]. Furthermore, due to economic constraints and limited resources, many countries are facing the reality of limiting the growth in the healthcare budget and spending. Constraints on healthcare budgets and the need of patient-centered care are the biggest problems concerning the developing and developed world [89]. The growth in increasing prevalence of healthcare problems, infectious diseases are leading to significant mortality and lifestyle diseases such as cardiac diseases and diabetes. Effective diagnostic testing has been difficult to achieve, as this requires regular monitoring and frequent blood test [90]. Thus, there is a rising demand of home based POC diagnostic devices which are defined as affordable, sensitive, specific, user friendly, rapid and robust, portable, and easy to use for sample introduction and interpretation [91]. While POCT is one of the most active segments within the diagnostic industry, and seen as an option to meet the need, the technological capabilities far outnumber the rate of POCT adoption. Accordingly, it is likely that POCT will expand significantly in the developed as well as developing countries in the next decade. Furthermore, it should also be kept in mind that as fast and practical POC’s technologies are, their specificity and sensitivity should not be compromised and it requires precise and rigorous research to optimized this tradeoff. Accurate and detailed health economic considerations are required to testify whether POCT approach encompasses cost effective devices and can be used in the cases where clear benefit have been demonstrated to patients [92,93,94,95].

### 3.2. Applications Qualified as POCT

Multiple areas of expertise, various elements and testing’s need to be combined in a place to introduce as a part of POCT, that includes rapid communication of assessment results in between patients and care provider, to ensure appropriate follow-up of patients and a system for fast-track initiation of treatments [96,97]. It should be perceived as consisting of diverse components and technologies together that can range from sophisticated to the most simplest form, having settings that can be utilized anywhere such as home, clinics, communities, peripheral laboratories as well as hospitals, it should be user-friendly and can be used by highly trained person to lay persons. In short, it should be a testing that can be done in diverse settings, ranging from hospitals to homes easily [98]. Many of the technologies are currently being qualified as POCT, a typical classification of POCT is subdivided into a small handheld devices includes qualitative and quantitative strips, and other one is more complex built-in fluidics a larger bench-top devices, generally the variants similar to the ones which are used in conventional laboratories. Although with the upcoming trend of increasing demand of miniaturization of devices along with the requisition of technologies expanded in the relation to consumer electronics, nowadays it is going to be increasingly possible to develop smaller devices that integrate all of the key design features [99,100]. Various common application such as: glucose monitoring kit, cholesterol/Lipids testing kit, food pathogen testing kit, pregnancy and fertility testing kits, urinalysis testing kit [101], tumor/cancer and cardiac marker testing kit, blood pressure measuring devices, hematology testing kit, fecal occult blood testing kit, coagulation monitoring kit, drug abuse testing, and infectious disease testing kits are some of the many applications qualified as POCT [102,103,104,105]. Other variable usages are also present in primary care setting such as creatinine, bilirubin, HgB A1c, heparin, magnesium microalbumin measurements, lipid profiling, HIV diagnostic kit [106], *H. pylori* diagnostic kit, PT/INR, D dimer, etc. Along with this sensor technologies for the rapid analysis of blood samples and biosensors technologies used to measure toxicology and drug screening are also available [107,108,109,110,111].

## 4. Quantum Dots as a Microsystems: Implication to POCT

The advent of nanotechnology has enabled the development of small-scale systems that can be used for varied biomedical applications like imaging, diagnostics and drug delivery. Development of microfluidics, or lab-on-a-chip technology is gaining pace within the biological research area and it sure is to simplify and revolutionize how we look at biological macromolecules as it offers several advantages such as decrease in sample and reagent consumption, multiple sample analysis, lesser time and high throughput analysis. A single chip can establish many processes like mixing, reaction, detection or separation within itself [112]. Therefore, microfluidic systems have allowed for integration of many bioprocesses and offered expansion of in vitro studies in research. The microfluidic devices are generally made with PDMS (polydimethilsyloxane) Sylgard and thin glass slides. PDMS is a non-toxic dimethyl silicone-based oil with low viscosity, high molecular weight, and distinctive flow properties. The most widely used methods for making a microfluidic device, with the help of PDMS and glass slides, are photolithography and replica molding technique [113]. These microsystems can be made as per the users’ choice of the number of chambers and microchannels. 

Researchers have used QDs based microfluidic systems in combination with biological imaging techniques for cytotoxicity studies [114]. A group used QD-IgG conjugates in combination with microfluidic protein chip for cancer detection and found that microfluidic systems improve the detection limit and sensitivity improves up to four orders in magnitude as compared to organic dyes [115]. Such small-scale integrated devices have been used for high throughput bioassays of DNA [116,117], bacteria [118,119], proteins [120,121], and viruses [122]. Various studies have been done in the development of immunoassays on a microfluidic chip that include detection of clinically important disease biomarkers such as C-reactive protein (CRP) [123], prostate specific antigen, ferritin, and vascular endothelial growth factor [124]. However, using Quantum dots as markers for detection of disease biomarkers and other important biomolecules still remains a gap, which can be further researched upon and utilized for development of microfluidic devices.

## 5. Application of QDs for POCT 

**Figure 1:**
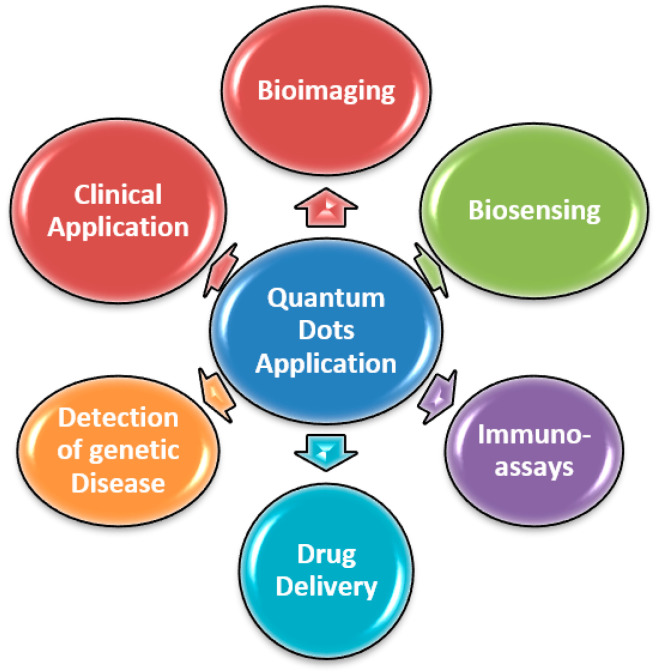
Different application of quantum dots.

QDs have demonstrated great potential and attracted intense investigation in biomedical and bioanalytical application due to their distinctive photoluminescence properties [125] and ability to fabricate them as biocompatible system with conjugation to varied biomolecules. Their application is underway in developing various molecular and immunological assays for different biomarkers and pathogens. Additionally, utilization of QDs in combination with their distinctive optical properties and microfluidics would assist in the development of sensitive bioanalysis system for their use in point of care testing. The easy availability of different type of QDs for clinical utility could likely see a recognizable advancement in their use. Thus, in the current review we will summarize the present development available in literature in context to the use of QDs for the aforementioned POCT including bioimaging (in vitro, live cells, in vivo and single molecule imaging) biosensing (protein DNA, immunoassay, and sugar sensing), bio-targeting (drug delivery, detection of genetic diseases, clinical application) [126] (Figure 1). 

### 5.1. Bioimaging Applications of QDs

Various imaging techniques such as optical imaging, magnetic resonance imaging and nuclear imaging are utilized as a key imaging technique in the biological systems and are complementary to each other. In spite of this, these techniques are high costs as well as requiring relatively long imaging time and are poor in their prognosis at early stage detection with several other drawbacks associated with their use [127]. Thus, there is a need to design a probe that can overcome these entire barriers. QDs application is evidence as excellent candidates for multimodal imaging probe and growing exponentially in medical as well in industry domain [128]. Conventional bioimaging which is based on biological species conjugated with organic dye to enhance the target, suffers big disadvantages of high photosensitivity, broad emission bands, narrow absorption, and sensitivity to media conditions [129,130,131] while on the other side QDs are propitious candidates and by controlling their size, composition, and coating one can tune the desirable emission as well as absorption wavelengths from ultraviolet to the near IR luminescent region [132,133]. They have very high quantum yield and signal high fluorescence brightness with the advantages of stimulating multicolor florescence by the same light source, with the very slightest spectra overlap, therefore providing significant advantages for multitarget bioimaging. Thus, QDs are the promising labels and can be utilized as an enhanced targeting probe in the area required multi color detection or high fluorescence [134]. Nevertheless, there are many studies with examples of QDs based conjugate labels used as bioimaging modalities. Therefore, in this review, we will first elaborate the application of QDs as bioimaging at the cellular and molecular level and then at the organ or tissue level to achieve anatomical and structural information [135,136].

#### 5.1.1. QDs Application in In Vitro Imaging and Analysis

QDs impart the best compromise between size and photophysical properties for the analysis and imaging of cells. Having size ranging from 2 to 20 nm diameter [137] makes QD’s a unique metallic and smallest nanoparticle with 200–10,000 atoms [138], produces enough electron density and high molar extinction coefficients and leads to high contrast in bioimaging [139]. Due to the surface functionalization properties QDs are biocompatible, stable and soluble in the biomatrix. Conjugation of surface coating of QDs let them allow to effectively incorporating by cells and are utilized as tags and labels for the analysis of cells in different fields such as intracellular cargo transport, drug and gene delivery, extracellular receptors, membrane dynamics, embryonic development, and cancer cell. They can utilized for the visualization of cellular receptor and structure in both live and fixed cells and thus mimic the properties of organic fluorophores along with various other advantages. Having advantages of increased photoluminescence, broad excitation spectra, increased photostability, and photoluminescent properties make them suitable for various immunohistochemical applications. QDs application as immunofluorescent, first demonstrated in 1998 by conjugating TGA-capped QDs by EDC-coupling with IgG where agglutination of QDs induced by antibody conjugated with the human IgG were first time clearly observed [140,141]. Thus, opening the way for future research related to application of QDs as immunofluorescent labeling. The QD-antibody conjugation utilizes two different approaches. In one approach, it involves QD’s mediated antigen labeling of both primary and secondary antibodies where the primary antibody attached with the antigen is recognized by the biotinylated secondary antibody, which is further attached to a streptavidin-coated QDs. The other approach consists of a biotinylated primary antibody conjugated with the streptavidin-coated QDs and directly recognized by the targeted antigen. Further QDs are also used as QD-micelles as a superior photostability fluorescent labels and a cell tracker during embryogenesis [142]. They are constricted to the injected cells and their individual daughter cells and their translocation into the nucleus cells can be observed during the embryo development at a particular stage. Recently, QDs are also utilized as a fluorescent label of cells in the study of the embryo development of Zebrafish [143,144].

#### 5.1.2. In Vivo Imaging

QDs are used as a fluorescent probe for the imaging of whole body, however due to the toxicity of QDs relatively less work has been done [145,146,147,148,149,150]. Interestingly with the development of fabrication technique of QD many researchers tried to explore the potential of QDs in broader area. A successful labeling of pig lymph nodes performed by using NIR-emitting QD probe technique has initiated a new challenge in the field of QD in vivo application. Systematic evaluation of distribution of QDs their absorption level, metabolisms, toxicity, and excretion have been carried out for different type of QDs. QDs based probes are also developed as a multifunctional nanoparticle in the field of cancer imaging in live animals [147]. Voura and group have observed that QD labeled tumor cells of lung tissue behaves indistinguishable in comparison with the unlabeled cells in mice when observed by spectral imaging [151]. Further cancer related antibodies coupled with the near-IR based QDs along with the polymer coating are one of the most approved QDs agents for the imaging of targeted tumor [152,153]. Kobayashi and group have reported for first time the trafficking of lymph node with simultaneous imaging, i.e., lymphangiography fluorescence by injecting five QDs having different emission spectra [154]. Although application of QD in in vivo imaging is spreading wider, the depth of targets makes the in vivo imaging of QDs very challenging and requires high sensitivity, low toxicity, photostability, and higher contrast.

### 5.2. QD’s Application as Biosensors

QDs are also excellent candidates of biosensor as they have unique property of attaching various biomolecules [155] covalently or non-covalently to their surface, which further capped QDs through cross-linker molecules without negatively affecting the properties of the molecules. The reactive groups present on the hydrophilic surface of the QDs aid in the attachment. The biomolecules such as oligonucleotides or serum proteins get adsorbed on the surface of hydrophilic QDs. Ionic strength, surface charge, pH, and temperature are some of the factors on which adsorption depends. Non-covalent interactions include the displacement of some capping agents by biomolecules like immobilization of the thiolated oligonucleotides on mercaptoacetic acid coated QD [156]. Various crosslinkers are used to link the functional groups like 1-ethyl-3- (3-dimethylaminopropyl) carbodiimide (EDC), which link –NH2 and –COOH groups [157]. 

Since streptavidin has a higher binding affinity for the biotin, the QDs are covalently linked to the streptavidin through carbodiimide-mediated coupling reaction. The biotin is attached to the biomolecules of interest. The conjugate of streptavidin and the conjugate of biotin are then used for the specific detection of antibodies, DNA, etc. [158]. There are several advantages for using QDs over fluorescent dyes, which includes higher quantum yield, higher photostability, and size-tunable fluorescence emission. The QD based sensors are highly specific, sensitive and stable [159]. Xiao and his coworkers showed the detection of human metaphase chromosomes by fluorescence in situ hybridization (FISH) using biotinylated DNA probe and QD-streptavidin conjugates which is useful for research and clinical applications [160]. Hahn et al. described the detection of pathogenic bacterial cells *Escherichia coli* O157:H7 serotype using CdSe/ZnS core/shell QDs conjugated to streptavidin and specific biotinylated antibodies which will be useful in the food industry [161]. Lee and his coworkers described the detection of transmissive stage, oocytes of the waterborne pathogen *Cryptosporidium parvum using* QD605-streptavidin together with biotinylated anti-*Cryptosporidium* sp. MAb, which is useful for public health safety in relation to the drinking water supplies [162]. Several ligands or peptide hormones can also be conjugated to the QDs which bind to the receptor in the living cells as shown by Tomlinson et al. where Angiotensin II (Ang II) was attached to AMP coated CdSe/ZnS core–shell nanocrystals through an 1-[3-(Dimethyamino) propyl]-3-ethylcarbo diimide hydrochloride (EDC) coupling [163]. The Chinese hamster ovary (CHO) cells which expresses angiotensin I were then fluorescently imaged by incubating with peptide–quantum dot conjugates. The DNA molecules can also be attached to the QDs, which can be used for the detection of the target DNA. Shi and Ma showed the conjugation due to the electrostatic interaction of the CdS nanocrystal having negative charge with the amidocyanogen DNA molecules having positive charge [164]. This CdS conjugated probe DNA then labels the target DNA that is immobilized on the quartz slides and helps in improving the quantitation analysis of DNA. They used CdS nanocrystals as it shows dramatic increase in the emission intensity as compared to CdSe, ZnS nanocrystals.

Since QDs have broad excitation spectra and narrow emission spectra, these are excellent donors for fluorescence resonance energy transfer (FRET)-based biosensors. Several FRET biosensors having QDs and dye-labeled molecules have been developed. CdSe/ZnS DNA conjugates were used in FRET for hybridization and DNA cleavage by Gill et al. Peng et al. developed the detection of DNA hybridization using blue luminescent QDs and dye labeled ssDNA [165]. Electrostatic linker was used between CdTe QDs and dye labeled ssDNA and this conjugate was then incubated with the sample DNA. Depending on the differential interaction with the sample DNA results into the differential changes of FRET efficiency. Zhang et al. reported the more rapid and sensitive method for the detection of nucleic acids by using two-color quantum dots, based on single molecule coincidence detection [166]. In comparison to conventional QD based assay, this method has high detection efficiency and high hybridization efficiency which results in short analysis time compared to conventional QD based assay. In this method, using sandwich hybridization reaction, two biotinylated oligonucleotide probes detect specific complimentary target DNA. The one end of this hybrid then through streptavidin biotin complex bind to the 605 QDs and then on adding 525 QDs binds to the other end of the hybrid forming 605 QD/DNA hybrid/ 525 QD complex and results in the homogenous, rapid, simple, and sensitive method for genomic DNA analysis.

### 5.3. Clinical Application of QDs 

QDs evident many clinical applications in the area of tracking different particles, labeling cells, work as imaging and drug delivery agent act as photodynamic therapy agents in terms with neurosciences. They are more than 100 times stable and 20 times brighter in comparison to the traditional reporters inducing fluorescence. They act as promising antimicrobial agents as well as novel sensor for antigens and allergens and used for the detection of protein. [167] They are very good fluorescent sensing platform for the detection of DNA. QDs amphipol and CdSe technology is used for both intracellular as well as real time imaging of siRNA delivery with less cytotoxicity into cancer cells. QDs are also utilized in tissue engineering to enhance the osteoblast production and reduce the chances of rejection by creating QDs on the surface of knee/hip prosthesis. They are used as a tracking agent for neural and ganglionic interaction. Having long blood circulation time and larger drug loading capacity they can easily be utilized for the controlled drug release profile, and integration of multiple targeting ligands on surface at a molecular level [168,169]. QDs are also utilized for imaging cellular neuronal membrane potential by directly measuring the electric field within the plasma membrane or by using FRET along with a proximal dye quencher/acceptor [170,171,172].

Further lateral flow test, is the point of care test, which is most common and is used for the detection of various analytes like nucleic acid, antibodies, biomarkers, viruses, etc. They are easy to use, give rapid result and are portable as compared to laboratory-based tests. Generally, colloidal gold is used in the lateral flow tests but due to the limited sensitivity and cost, QD based lateral flow test are developed which are economical, are highly sensitive and gives the rapid result apart from the other advantages it has as mentioned earlier. Yang et al. described the novel QD-based point of care test for syphilis [173]. Staphylococcal protein A (SPA) is conjugated to the QD. The QD labeled SPA is mixed with the sample and the QD based lateral strips are then inserted into this solution. These strips contain one control line, which contains IgG and one test line, which contains the mixture of TP15, TP17, and TP47 recombinant syphilis antigen. Thus, QD-based point of care test is suitable for the rapid on-the site screening of syphilis. Cheng and his coworkers described the QD and immunochromatographic test strips assay for the rapid and quantitative detection of C-reactive protein (CRP) [174]. CRP is the protein, which is the inflammation marker and helps in the risk assessment of the cardiovascular diseases. This assay uses the double antibody sandwich method. The immunochromatographic strip has control line, which has rabbit IgG and test line, which has human CRP antibody. The fluorescence immunochromatographic solution contains QD-labeled goat anti rabbit antibody and QD-anti CRP antibody, which are mixed with the samples. On applying the sample on the strip, rabbit IgG antibody captures QD-labeled goat anti rabbit antibody on the control line whereas human CRP antibody captures CRP forming (QD) Ab1–Ag–Ab2 complexes on the test line and then the fluorescence intensity is calculated. Similarly, Zhou et al. described the faster QD POCT fluorescence immunoassay detection method for high-sensitivity cardiac troponin [175]. High-sensitivity cardiac troponin is a biomarker of myocardial injury and necrosis. This assay also uses the double antibody sandwich method and accuracy, and sensitivity was determined by this method.

To make the QDs hydrophilic, the organophilic surface molecules are exchanged with polar molecules. However, these are subjected to degradation by hydrolysis or oxidation of capping ligands. Therefore, incorporating QDs into polymer microbeads has made more advancement and is further useful for multiplexed immunoassays. Nie et al. synthesized the chitosan nanoparticles with the QDs embedded in it with the help of amine groups present on chitosan and carboxyl groups on QDs [176]. This leads to the encapsulation of multicolor QDs and the quantum yield of the embedded QDs is higher than the free QDs. Wang et al. reported the sensitive detection of hepatitis B virus through dot immunoassay using QD nanobeads (QDNBs) could detect very low amount of hepatitis B surface antigens [177]. Hepatitis B antibodies can be conjugated to these nanobeads followed by incubation with the immobilized hepatitis B surface antigens on porous polyvinylidene difluoride.

Recent advances have been made by recombinant DNA technology by which recombinant protein linkers can be produced that contains basic leucine zipper fusion proteins that provides a net positive charge. The fusion partner is a protein domain of interest, the activity of which can be probed by the negatively charged QDs and is bound to the linker through electrostatic interactions. Mattoussi et al. reported the method of conjugating maltose-binding protein (MBP) to the lipoic acid capped CdSe/ZnS QDs by leucine zipper motif. The photoluminescent properties of QD were enhanced in the conjugate [178]. These applications have been tabulated in Table 1.

## 6. Challenges for the Use of QDs for Diagnosis

QDs are expanding in a range of biomedical application, but are still restricted to the basic research in spite of their significant progress and the obvious potential, due to their biocompatibility, short-term stability, chronic toxicity, and long-term breakdown. The level of cytotoxicity determined by various factors occur from inherent physicochemical properties as well as environmental conditions such as size, color, dose of QDs, surface chemistry, capping materials, processing parameter and coating bioactivity [179]. Along with this, due to the toxicity of QDs they are restricted to the selected in vivo studies and limit their application range to work in the field of biomedical uses [180]. Some studies even suggested that releasing of Cd2+ ions could not be prevented even after robust coatings of the surface of QDs and thus it required enhancing safety level before their use as a fluorescent probe in a biological application. Furthermore, it has been also found that engineered QDs are not considered as a uniform group of substances and most of the applications occur in bulk solutions thereby limiting the development of reusable sensors. Due to their known high toxicity, the routine analytical applications are also hindered so far [160]. CdSe QDs under UV irradiation for a prolonged time are highly toxic to the cultured cells due to the release of CD+2 ions during photolysis and can harm the cells. A number of notable difficulties prevent global uptake of the QD technology at present. The big size of QDs compared to currently present fluorophores affects their capability to examine and label the molecules of cells and might reduce the tissue penetration on a larger scale. There is still an uncertainty related to toxicity of QDs in vivo specifically related to their distribution and breakdown process and thus precludes the use of QDs in POCT in human application. It is also found that various assays incorporated QD, specifically based on immunofluorescence are reported as less sensitive compared to other assays. Although this is still not known and requires further investigation that the less sensitivity of QDs is because of the antibody used or assay design or due to the addition of QDs itself. In addition, the fundamental characteristics of QDs physiochemical properties and surface chemistry are poorly understood in varying situations which creates significant concerns for the potential use of QDs as POCT and require to be fully addressed. Therefore, it is important to explore out new synthesis methods to reduce the risk factor and coat them well so they are biologically inert. Other problems related to the QDs are that they have a very poor stability under ambient condition, so new methods have to be developed which can improve the stability of it in solution and the bench lifetime of QDs should be explored [181,182]. 

## 7. Future Prospect

A pervasive trend in the micromachines used in point of care testing is to develop high throughput and ultrasensitive technology for the fast-track detection, quantification and diagnostic. QDs are emerging as a specialized micromachine in the field of POCT and enable a new avenue of research because of their unique surface and structural properties such as wide spectrum band, tunable size, large surface-to-volume ratio, and stability. QDs based biomedical and pharmaceutical technology will be constantly expanding its application in POCT field. By utilizing QDs, various useful outcomes have been generated and are particularly advantageous, specifically in the area of single molecular tracking by using their photostability and long fluorescence lifetime. For the stable, stoichiometrically well-defined QD-conjugates, the mild and selective coupling of QD and biomolecules is in great demand. New methods may be developed for increasing the quantum yields of the QDs. Attachment of multiple functional groups to QD at the same time will be beneficial for combined array based detection systems. Better carriers and increased sensitivity of QDs will result in better-targeted delivery [9]. Further QD and biomolecule conjugates utilized for in vitro and in vivo imaging are opening avenues for future research, which can focus on the formation of QDs conjugation for cancer diagnosis and cell labeling. The capability of tuning the size of QDs is favorable and advantageous for various applications. Due the larger surface area of QDs it can covalently bond to bio recognition molecules such as peptides, antibodies, small molecule ligands or nucleic acids and used in an application as fluorescent probes. Numerous useful findings have been generated by using QDs properties such as photostability and their long lifetime of fluorescence especially in the area of tracking of single-molecules, QDs sensors for the visualization of single living cell surface charge. Another area involving multiplex imaging, QDs can provide perfect probe and open an opportunity of significant progress in various field. Interestingly, novel routes will also be opened for the synthesis of nanocrystals if the enzymatic procedures can be combined with the bioorganic synthesis methods. QDs can be utilized in future to promote personalized clinical treatment based on the molecular profiles of every patient individually. In addition, for the production of more suitable biocomponents, more genome and proteome studies are [183] required where QDs can be incorporated leading to detection of diverse disease biomarkers. There have been postulations over plausible use of QDs in a wide number of applications, but care should be taken as a number of concerns have yet to be answered and the behavior of QDs is yet to be completely characterized. Finally, QDs research requires new innovations and versatility in the development of QDs, which are free from nonspecific adsorption, low toxicity, monovalent, multiplex functionalities, having new surface coating, cellular delivery strategies, molecular tagging, and compact in size with enough brightness to be utilized in a single cell imaging [184,185].

## 8. Conclusions

This article summarizes a rapidly increasing research field of application of QDs in point of care testing. It establishes the fundamental principles of using QD as fluorescent probe along with the published examples. The unique optical and electronic properties of QDs are already meeting the challenges in the developing field of nanotechnology. Considerable effort has been made to improve the biological properties of QDs by polymer encapsulation to increase the quantum yield, to hydrophilicity, aggregation, and bio-distribution. The biological properties are exploited for the detection of various biomolecules, the methods of which are continuously evolving to give the best results. Though there are several methods to conjugate the functionalized nanocrystals with biomolecules, there is a great requirement of mild and selective coupling techniques which will result in the thermodynamically stable, stoichiometrically well-defined QD-conjugates and therefore more careful and elaborate research will be necessary. However, the dramatic developments in this field are successful so far and a similar rate of progress will promise plenty of excitement from future innovations [186].

## Figures and Tables

**Table 1:** Various applications and advantages of biofunctionalized quantum dots.

S. No.	Recognition Moiety Conjugated to QD	Target Analyte	Application	Advantage	References
1.	Streptavidin	Biotinylated total human DNA probe in metaphase chromosomes	For pathology and medical genetics diagnostics	Photostability and high sensitivity	[160]
2.	Streptavidin	Biotinylated anti-*E. coli* O157:H7 antibodies	Detection of pathogenic *E.Coli* O157:H7 cells	Highly selective, sensitive and stable sensor as compared to other conventional methods like polymerase chain reaction (PCR) analysis, enzyme-linked immunosorbent assays (ELISAs), and fluorescence-based assays using organic dye molecules	[161]
3.	Streptavidin	Biotinylated mab	Detection of transmissive stage of waterborne pathogen, *Cryptosporidium parvum* oocysts	High photobleaching threshold of quantum dots in comparison to the use of inorganic fluorophores	[162]
4.	Angiotensin II	Incubated with the Chinese hamster ovary (CHO) cells	Imaging the live Chinese hamster ovary cells that expresses angiotensin II type I receptor	Higher quantum yield and higher photostability as compared to the angiotensin dye conjugate	[163]
5.	CdS QD with carboxyl group	DNA molecules	Labeling DNA molecules	The quantitation analysis of DNA is improved due to the use of stable QD fluorophores	[164]
6.	Adenine and AMP	Incubated with bacteria	Labeling bacteria with Purine dependent mechanisms	Can be used to probe the interactions between ligands, membrane proteins, cell wall	[145]
7.	QDs-labeled Staphylococcal Protein A (SPA)	QD based test strip containing the mixture of TP15, TP17 and TP47 recombinant syphilis antigen as a test line.	A novel QD-based point of care test for syphilis	Lateral flow test, which is inexpensive, sensitive, and easy to use	[170]
8.	One monoclonal CRP antibody conjugated to QD (signal antibody)	Other mouse monoclonal CRP antibody which is a capturing antibody is used to capture CRP antigen to form QD (Ab1–Ag–Ab2 complexes on the test line)	Used for the detection of C-reactive protein (CRP), the levels of which are used for the risk assessment of cardiovascular diseases	High-sensitivity and photostable as compared to other conventional immunochromatographic strips	[171]
9.	One monoclonal cardiac troponin antibody conjugated to QD (signal antibody)	Other mouse monoclonal cardiac troponin antibody which is a capturing antibody and is used to capture cardiac troponin antigen to form (QD) Ab1–Ag–Ab2 complexes on the test line	Used for the detection of cardiac troponin which is a biomarker of myocardial injury	High fluorescence efficiency, high photostability, and easy surface modification	[172]
10.	Cy3-ssDNA	ssDNA or dsDNA	DNA hybridization detection based on FRET	In this method of solution-based fluorescence detection minimal DNA modification is required	[165]
11.	One end of DNA hybrid (two biotinylated oligonucleotide probe bound to specific complementary target DNA) bound to Streptavidin conjugated 605QD	Streptavidin conjugated 525QD bound to the other end of the DNA hybrid to form the 605QD/DNA hybrid/525QD complex	Homogenous rapid and sensitive nucleic acids detection method using two-color quantum dots (QDs)	High detection efficiency, suitable for temperature treatment and short analysis time due to the increased hybridization efficiency because of high diffusion coefficient	[166]
12.	3-mercaptoropionic acid	Chitosan nanoparticle with embedded QD	Bioassays and intracellular labeling	Small size and higher fluorescence quantum yield	[173]
13.	Hepatitis B antibodies conjugated to Quantum dot nanobeads (QDNBs)	Hepatitis B surface antigens immobilized on the porous polyvinylidene difluoride	Used for the detection of Hepatitis B Virus through dot immunoassay	High sensitivity which can detect as low as 78pg of hepatitis B surface antigen	[174]
14.	Lipoic acid	Recombinant fusion protein comprising leucine zipper domain and maltose binding protein domain	To probe the interactions between the maltose binding protein (MBP) and an amylose affinity resin	Higher quantum yield	[175]
